# A rare case of proliferative glomerulonephritis with monoclonal IgG2 kappa deposit: a case report

**DOI:** 10.1186/s12882-022-03029-6

**Published:** 2022-12-09

**Authors:** Daisuke Ito, Yuriko Shiozaki, Yoshitaka Shimizu, Yumiko Suzuki, Asami Takeda, Taro Misaki

**Affiliations:** 1 Division of Nephrology, Seirei Hamamatsu General Hospital, Hamamatsu, Shizuoka, Japan; 2 Division of Nephrology, Japanese Red Cross Aichi Medical Center, Nagoya Daini Hospital, Nagoya, Aichi Japan

**Keywords:** Proliferative glomerulonephritis with monoclonal immunoglobulin G deposits, Membranoproliferative glomerulonephritis, IgG2 kappa

## Abstract

**Background:**

Proliferative glomerulonephritis with monoclonal immunoglobulin G (IgG) deposits (PGNMID) is a rare monoclonal gammopathy of renal significance with dense deposits of monoclonal immunoglobulin.

**Case presentation:**

We report a 78-year-old Japanese male patient with mild proteinuria and lower extremity edema. Monoclonal immunoglobulin could not be identified in his serum or urine. Although his bone marrow biopsy was negative, renal biopsy found features of membranoproliferative glomerulonephritis (MPGN) with deposition of monoclonal IgG2 kappa. Electron microscopy examination revealed non-organized electron-dense deposits in the subepithelial, and subendothelial mesangial regions. Steroid monotherapy was performed after diagnosis of PGNMID but complete remission was not achieved.

**Conclusion:**

PGNMID with IgG3 kappa deposits is the most common in cases with the histological feature of MPGN. There are few cases of PGNMID with IgG2 kappa deposits exhibiting MPGN. This report describes a very rare case of PGNMID with the histological feature of MPGN.

## Background

Proliferative glomerulonephritis with monoclonal IgG deposits (PGNMID) is a rare disease that is regarded as one subtype of monoclonal gammopathy of renal significance [1]. It is reported that the renal biopsy incidence of PGNMID is only about 0.17% [1]. Clinically, patients with proteinuria and microscopic hematuria gradually develop renal dysfunction. They have renal pathology including endocapillary proliferative, membranoproliferative or membranous features with positive staining of glomerular IgG or IgA deposits, which are restricted to a single IgG or IgA subclass [1, 2]. The efficacy of immunosuppressive agents, such as steroids, cyclophosphamide, cyclosporine, mycophenolate, bortezomib, rituximab or daratumumab, for treatment of PGNMID has not been established [3–5].

Here, we present a case of PGNMID that was treated with steroid monotherapy. The biopsy revealed PGNMID with features of membranoproliferative glomerulonephritis (MPGN), characterized by the presence of monoclonal IgG2 kappa and non-organized electron-dense deposits (EDDs), which are located in the subepithelial, and subendothelial regions. This report describes a very rare case of PGNMID with IgG2 kappa deposits exhibiting MPGN.

## Case presentation

A 78-year old man with type 2 diabetes mellitus and hypertension presented with mild proteinuria, lower extremity edema, and worsening renal function. His serum creatinine level was 1.22 mg/dL just before referral to our hospital. His type 2 diabetes mellitus was well controlled for several years (hemoglobin A1c 6.2–7.4%) and there was no family history of kidney disease. On admission to our hospital, his blood pressure was 132/48 mmHg, pulse rate 78 beats/min and body temperature 36.9 °C. Physical examination revealed no significant systolic heart murmur on auscultation. Examination of the lungs, abdomen, and central and peripheral nervous systems found no abnormalities. However, mild edema was observed in the lower extremities. The patient’s laboratory data on admission are summarized in Table 1. Urinalysis showed markedly elevated proteinuria (protein excretion, 6.37 g/day) and hematuria (10–19 red blood cells/high-powered field). Serum albumin level was 3.1 g/dL, and serum creatinine level was 1.68 mg/dL (reference range, 0.65–1.07 mg/dL), with estimated glomerular filtration rate of 56 mL/min/1.73 m^2^. White blood cell count was 8.62 × 10^3^ cells/μL (reference range, 3.3 × 10^3^–8.6 × 10^3^ cells/μL), hemoglobin level was 10.5 g/dL (reference range, 13.7–16.8 g/dL), and platelet count was 22.4 × 10^4^ cells/μL (reference range, 15.8 × 10^4^–34.8 × 10^4^ cells/μL). Serum IgG level was 1120 mg/dL (reference range, 861–1747 mg/dL), IgA level was 222 mg/dL (reference range, 93–393 mg/dL), IgM level was 28 mg/dL (reference range, 33–183 mg/dL), and IgE level was 539 U/mL (reference range, 0–360 U/mL). Both serum free kappa (64.7 mg/dL) and lambda light (46.9 mg/dL) chain levels were mildly elevated (reference range of free kappa level and free lambda light levels, 3.3–19.4 and 5.7–26.3 mg/dL, respectively) and there was no deviation. M-protein on serum immunofixation electrophoresis was negative. Bence–Jones protein on urine immunofixation electrophoresis was negative. Plasma C3 level was 95 mg/dL (reference range, 73–138 mg/dL), and C4 level was 27 mg/dL (reference range, 11–31 mg/dL). Hepatitis B surface antigen, and hepatitis C virus, human immunodeficiency virus, and human T-cell leukemia virus antibodies were all negative. The patient was positive for anti-nuclear antibodies but negative for anti-neutrophil cytoplasmic, anti-glomerular basement membrane and anti-DNA antibodies, and cryoglobulin (Table 1). His bone marrow biopsy exhibited absence of clonal B cells or plasma cells.

**Table 1:** Laboratory data

**Blood**			
White blood cells	8620/μL	IgG	1120 mg/dL
Hemoglobin	10.5 g/dL	IgA	222 mg/dL
Platelet count	22.4 × 10^4^/μL	IgM	28 mg/dL
Creatinine	1.68 mg/dL	Free κ chain	64.7 mg/dL
Urea nitrogen	35 mg/dL	Free λ chain	46.9 mg/dL
Total protein	6.2 g/dL	IgG1	603 mg/dL
Albumin	3.1 g/dL	IgG2	364 mg/dL
HbA1c	6.4%	IgG3	68.7 mg/dL
Blood glucose level	111 mg/dL	IgG4	121 mg/dL
Total cholesterol	219 mg/dL	C3	95 mg/dL
D-Dimer	2.5 μg/mL	C4	27 mg/dL
Anti-streptolysin-O	21 U/mL	CH50	58 U/mL
Anti-streptokinase	27 U/mL	T-SPOT	Negative
β-d-Glucan	10.6 pg/mL	Cryoglobulin	Negative
HBs-antigen	Negative	Rheumatoid factor	Negative
HBs-antibody	Negative	Anti-nuclear antibody	80
HBc-antibody	Negative	Anti-DNA-antibody	Negative
HCV-antibody	Negative	MPO-ANCA	Negative
HIV-antibody	Negative	PR-3-ANCA	Negative
HTLV-antibody	Negative	Anti-GBM antibody	Negative
CRP	0.23 mg/dL	Serum protein- electrophoresis	Normal-Pattern
**Urinalysis**			
Proteinuria	4+		
Protein	6.37 g/day		
Occult blood	3+		
Red blood cell	10–19/HPF		
Bence–Jones protein	Negative		
α1-Microglobulin	44 mg/L		
N-Acetyl-β-d-glucosaminidase	60.6 U/L		

*Abbreviations*: *CH50*, *CRP*, C-reactive protein, *GBM*, *HbA1c*, glycosylated hemoglobin *A1cHPF*, high-power field, *MPO-ANCA*, *PR3-ANCA*, T-SPOT

**Fig. 1:**
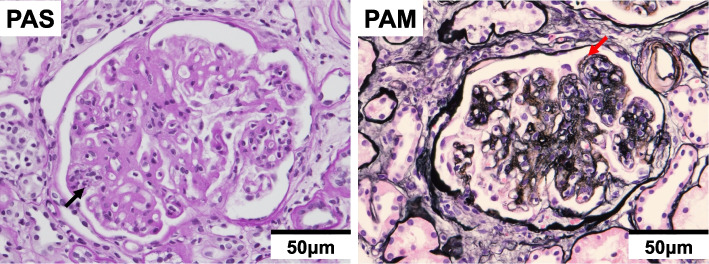
Light microscopy findings (PAS stain; PAM stain, 400x). Light microscopy Olympus BX53, OLYMPUS DP73 camera, and cellSense (Olympus Corporation, Center Valley, PA, USA) was used to capture the images. The images were obtained with eyepiece at 10X magnification and objective at 40X. No enhancement of the images was performed. The measured resolution was 4800 × 3600. The histological pattern was predominantly membranoproliferative glomerulonephritis characterized by diffuse and global double-contoured glomerular capillary walls (red arrow) with mesangial cell interposition and mesangial expansion by increased mesangial cell number and matrix. Moreover, it showed endocapillary hypercellularity (black arrow)

**Fig. 2:**
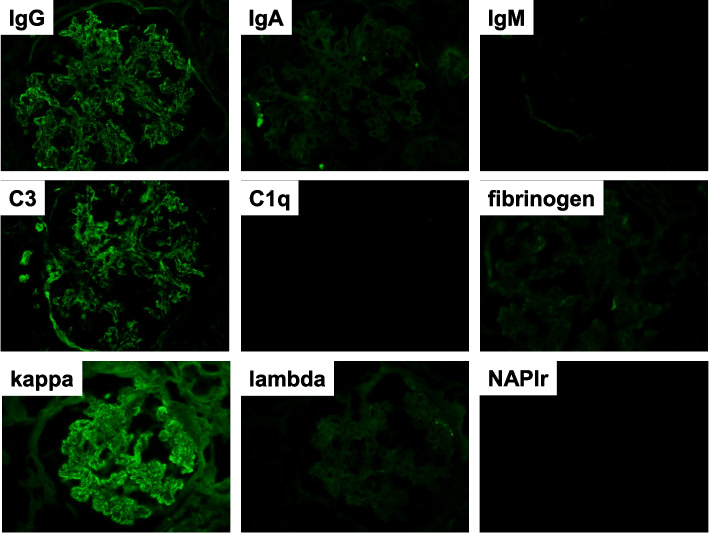
Immunofluorescence analysis was performed using fluorescein-isothiocyanate-conjugated antibody against IgG, IgA, IgM, C3, C1q, fibrinogen, kappa chain, lambda light chain or nephritis-associated plasmin receptor (NAPlr). Fine granular staining of IgG (2+), C3 (2+) and kappa chain (2+) was identified, along the glomerular capillary walls and in the mesangial areas, while IgA, IgM, C1q, fibrinogen, lambda light chain, and NAPlr were all negative (400x)

**Fig. 3:**
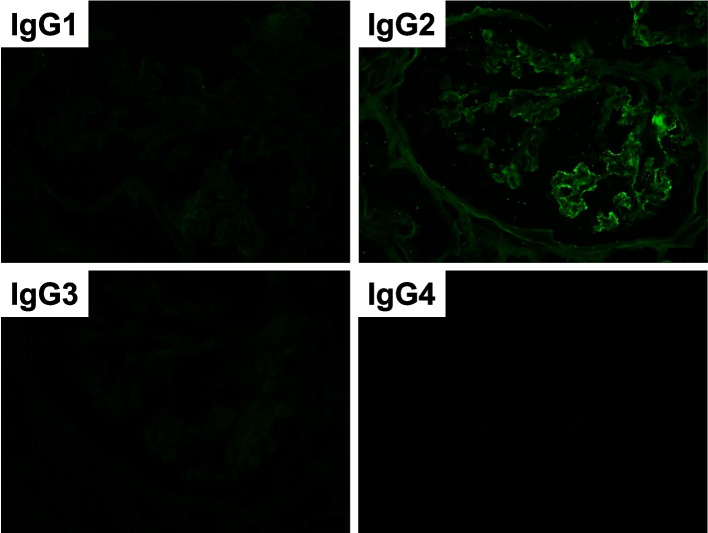
Immunofluorescence analysis was performed using primary antibodies against IgG1, IgG2, IgG3or IgG4 and secondary fluorescein-isothiocyanate-conjugated antibody. Fine granular staining of IgG 2 (2+) was identified, along the glomerular capillary walls and in the mesangial areas, while the other IgG subclasses (IgG1, IgG3 and IgG4) were all negative (400x)

**Fig. 4:**
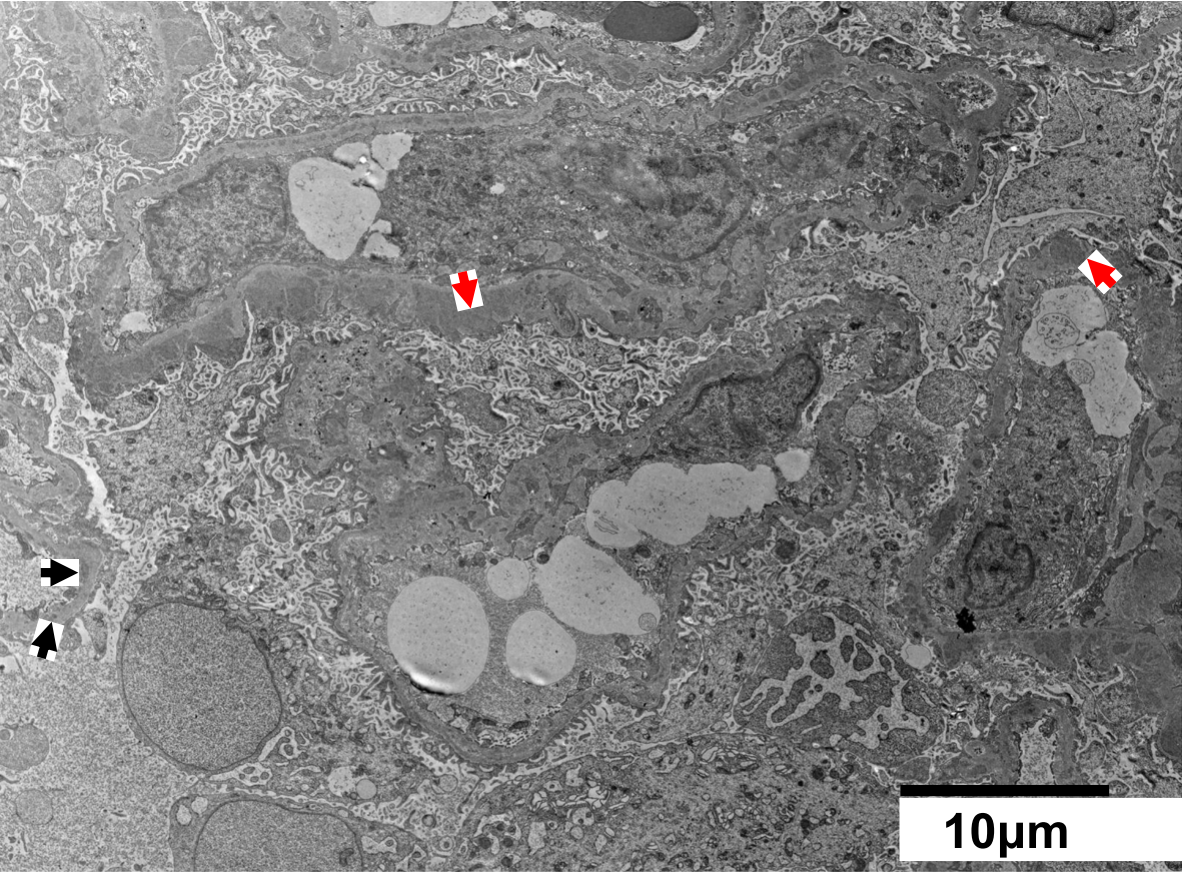
Electron microscopy revealed electron-dense deposits (EDDs; black arrows) located in the subepithelial (red arrow) and subendothelial (black arrow) regions. Non-organized structures could readily be identified within the EDDs

Based on the above clinical findings, percutaneous renal biopsy was performed to determine the pathological characteristics of the renal dysfunction. The biopsied sample consisted of a renal cortex containing 14 glomeruli, all of which were segmentally or globally non-sclerotic. The histological pattern was predominantly MPGN characterized by diffuse and global double-contoured glomerular capillary walls with mesangial cell interposition and mesangial expansion by increased mesangial cell number and matrix. Moreover, it showed endocapillary hypercellularity (Fig. 1). Masson trichrome staining showed some tubulointerstitial damage (data not shown). Direct first scarlet staining revealed no amyloid deposition (data not shown). Immunofluorescence produced strong glomerular staining of IgG and C3 globally in the peripheral capillary loops and segmentally in the mesangial areas (Fig. 2). The deposited IgG was composed only of subclass IgG2 kappa light chain (Figs. 2 and 3). Electron microscopy revealed that EDDs were located in the subepithelial and subendothelial regions. Non-organized structures were readily identified within the EDDs (Fig. 4). No EDDs were detected in the tubulointerstitium and arterial walls (data not shown). Based on the above histological and immunohistological findings, we diagnosed this case as PGNMID with monoclonal IgG2 kappa deposits exhibiting MPGN features.

**Fig. 5:**
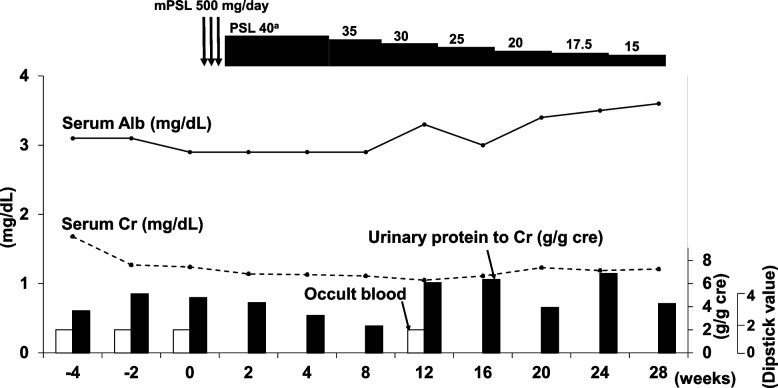
Clinical course of the patient. Abbreviations: Alb, albumin; Cr, creatinine; mPSL, methylprednisolone; PSL, prednisolone. PSL dose given in mg/day

Steroid pulse therapy (methylprednisolone 500 mg/day for 3 days) and subsequent oral administration of 40 mg/day (0.6 mg/kg) prednisolone were performed after diagnosis of PGNMID with monoclonal IgG2 kappa deposits. The prednisolone dose was gradually reduced to 15 mg/day over 6 months. Although proteinuria and serum creatinine level were not changed, serum albumin level gradually increased (Fig. 5).

## Discussion and conclusions

To our knowledge, this is a rare case of PGNMID with monoclonal IgG2 kappa deposit, exhibiting MPGN features. Renal biopsy revealed MPGN characterized by diffuse and global double-contoured glomerular capillary walls with mesangial cell interposition and mesangial expansion by increased mesangial cell number and matrix. Immunofluorescence revealed monoclonal IgG2 kappa deposits within the glomeruli, which was suggestive of PGNMID. Electron microscopy analysis showed non-organized EDDs in the subepithelial, and subendothelial regions.

Monoclonal gammopathy of renal significance (MGRS) is defined as hematological disorders with kidney disease [3]. Importantly, the B-cell or plasma-cell disorders of patients with MGRS do not demand any current hematological criteria for multiple myeloma and B-cell lymphoproliferative disorders. MGRS is commonly the consequence of the renal deposition of monoclonal immunoglobulins or their components either directly or indirectly. PGNMID is a rare disease, which was reported first by Nasr et al. [1]. They described three main characteristics of PGNMID: (1) endocapillary proliferative, membranoproliferative or membranous glomerulonephritis with glomerular IgG deposits, which are restricted to a single IgG subclass (IgG1–4) and a single light chain subclass (kappa or lambda); (2) the presence of non-orginized deposits by electron microscopy; and (3) exclusion of cryoglobulinemia [1]. Thus, PGNMID is classified as one subtype of MGRS [3]. Our case demanded the above three features. However, neither serum immunoelectrophoresis nor urine electrophoresis suggested monoclonal gammopathy. Moreover, the bone marrow biopsy exhibited absence of clonal plasma cells. Actually, approximately 70% of cases do not have detectable blood or bone marrow monoclonal immunoglobulins [4]. Thus, many PGNMID cases might not detect monoclonal immunoglobulins. A potential limitation about this is that a clonal small population could not be detected by the currently used techniques possibly. Another possible explanation is that the clone is located in the extra-medullary lymphoid tissue or organs [6].

The most common histological pattern of PGNMID is MPGN pattern, characterized by diffuse and global double-contoured glomerular capillary walls with mesangial deposits. The second most common type is endocapillary proliferative glomerulonephritis pattern, which is characterized by endocapillary hypercellularity and active inflammatory cell infiltration [1]. IgG3 kappa is the most common type of immunoglobulin in cases with the histological pattern of MPGN. Some reports show that about 50% of PGNMID patients have IgG3 deposition, and about 30% have IgG1 deposition [1, 7, 8]. Interestingly, there are few cases of PGNMID with IgG2 kappa exhibiting MPGN features, although there is one report of PGNMID with IgG2 lambda exhibiting MPGN features and another about PGNMID with IgG2 kappa exhibiting membranous nephropathy [9, 10]. Moreover, Bhutani et al. reported that PGNMID with IgG3 tended to have MPGN pattern, whereas PGNMID with IgG1 tended to show MN pattern [6, 11]. Regarding PGNMID with IgG4, there was one report about MN pattern [12]. The management of PGNMID is not well established. Thus, the treatment is based on some factors, including the presence or absence of monoclonal immunoglobulins and the risk for progression of kidney disease. Many patients have been treated with renin–angiotensin system (RAS) inhibition alone or with immunosuppressive agents including steroids, cyclophosphamide, cyclosporine, mycophenolate, bortezomib, rituximab or daratumumab, with variable success rates [3–5, 13]. Nasr et al. reported that only four of 32 patients with PGNMID achieved complete remission (reduction of proteinuria to < 500 mg/day). Many patients did not show significant recovery, although various kinds of therapies were administered. Only one of the six patients treated with steroid monotherapy had complete remission, and the others had incomplete remission, persistent renal insufficient or end-stage renal disease [1]. In our case, proteinuria and serum creatinine level were not changed, even though steroid monotherapy were administered. Additional therapies, including other immunosuppressive agents, might be necessary.

This is believed to be the rare case of proliferative glomerulonephritis with monoclonal IgG2 kappa deposition exhibiting MPGN features. Further studies on a larger number of PGNMID cases are required to confirm the association between IgG subclasses and clinical outcomes.

## Data Availability

All data generated or analysed during this study were obtained from the corresponding author on reasonable request.
